# Racial differences in microRNA and gene expression in hypertensive women

**DOI:** 10.1038/srep35815

**Published:** 2016-10-25

**Authors:** Douglas F. Dluzen, Nicole Noren Hooten, Yongqing Zhang, Yoonseo Kim, Frank E. Glover, Salman M. Tajuddin, Kimberly D. Jacob, Alan B. Zonderman, Michele K. Evans

**Affiliations:** 1 Laboratory of Epidemiology and Population Sciences, National Institute on Aging, National Institutes of Health, Baltimore, MD 21224, USA.; 2 Laboratory of Genetics and Genomics; National Institute on Aging, National Institutes of Health, Baltimore, MD 21224, USA

## Abstract

Systemic arterial hypertension is an important cause of cardiovascular disease morbidity and mortality. African Americans are disproportionately affected by hypertension, in fact the incidence, prevalence, and severity of hypertension is highest among African American (AA) women. Previous data suggests that differential gene expression influences individual susceptibility to selected diseases and we hypothesized that this phenomena may affect health disparities in hypertension. Transcriptional profiling of peripheral blood mononuclear cells from AA or white, normotensive or hypertensive females identified thousands of mRNAs differentially-expressed by race and/or hypertension. Predominant gene expression differences were observed in AA hypertensive females compared to AA normotensives or white hypertensives. Since microRNAs play important roles in regulating gene expression, we profiled global microRNA expression and observed differentially-expressed microRNAs by race and/or hypertension. We identified novel mRNA-microRNA pairs potentially involved in hypertension-related pathways and differently-expressed, including *MCL1*/miR-20a-5p, *APOL3*/miR-4763-5p, *PLD1*/miR-4717-3p, and *PLD1*/miR-4709-3p. We validated gene expression levels via RT-qPCR and microRNA target validation was performed in primary endothelial cells. Altogether, we identified significant gene expression differences between AA and white female hypertensives and pinpointed novel mRNA-microRNA pairs differentially-expressed by hypertension and race. These differences may contribute to the known disparities in hypertension and may be potential targets for intervention.

Hypertension is a major cause of death and disability worldwide. In the United States, almost 30% of the US population over age 18 have hypertension; however, African Americans (AA) have the highest prevalence of hypertension and cardiovascular disease (CVD) in the United States and have higher mortality attributed to CVDs than whites^1^. Heart disease and stroke are the first and third leading causes of death in women in the United States^2^, respectively, and AA women ≥20 years old have a higher prevalence of hypertension than white women (47% to 31%)^1^. AA women also have higher mortality attributed to cerebrovascular diseases than white women and AA women are more likely to have uncontrolled hypertension^3^. The underlying causes of AA predisposition to hypertension are still relatively unknown, particularly in women.

Previous studies have identified a handful of hypertension-related differences between AAs and whites. For example, hypertensive AA men and women have increased circulating inflammatory endothelial cells (CIECs) compared with whites^4^. CIECs detach from the endothelial layer at sites of tissue injury and may play a role in hypertension by increasing inflammatory states^4^. 46 genes in endothelial cells, found to be associated with shear stress response, are elevated in healthy AAs compared to whites and this may influence vascular and endothelial function^5^. In peripheral blood mononuclear cells (PBMCs), healthy AA men have higher mRNA levels of angiotensin II type I receptor (*AGTR1*) than healthy white men and exhibit increased production of superoxide, potentially contributing to oxidative stress and inflammatory states in healthy AA men^6^. These studies are among the few that examine gene expression differences which may play a role in racial hypertension disparities, but they are limited as they do not investigate the regulatory mechanisms governing these observed differences. Examining differential gene expression in the groups most affected by hypertension, such as AA women, may provide the best insight into understanding hypertension disparities^1^. Additionally, identifying the mechanisms contributing to these differences may help elucidate novel therapeutic targets for these at-risk populations and mechanisms governing response to hypertension treatment^7^.

MicroRNAs (miRNAs) are short, single-stranded RNAs that post-transcriptionally repress gene expression by either inhibiting translation or causing mRNA degradation of target genes^8^. miRNAs are critical regulators of gene expression in the cardiovascular system, playing an essential role in the regulation of cardiomyocyte proliferation^9^, signaling between circulating mononuclear cells and the microvascular endothelium^10^,^11^, and signaling between endothelial cells and the underlying smooth muscle cells^12^. Regulation of target genes by miRNAs influences cardiovascular function and health. For example, inhibition of macrophage-expressed miR-155 reduced cardiac hypertrophy and inflammation in mice, mediated in part through altered regulation of suppressor of cytokine signaling 1 (Socs1)^13^. miR-19b regulates cholesterol transport in macrophages by repressing the expression of ATP-binding cassette transport A1 (ABCA1) and promoting foam cell accumulation in atherosclerotic lesions in aortas of mice^14^. Endothelial expression of miR-126-5p changes in response to shear stress and disturbed laminar flow by regulating Dlk1, thereby influencing endothelial cell proliferation in regions of vascular injury^15^. Lower levels of miR-376c in platelets were observed in AAs compared to whites and were found to contribute to increased PAR-4 mediated-platelet aggregation via targeting of *PCTP*^16^. This differentially-altered pathway may affect response to anti-thrombotic therapies dependent upon race^16^. However, there have been no comprehensive analyses of gene expression in hypertensive women of different races or any studies identifying regulatory mechanisms contributing to differential gene expression in AA and white hypertensive women.

Few studies have examined differential mRNA and miRNA expression in the context of hypertension. One study identified five differentially-expressed miRNAs in PBMCs from hypertensives which correlated with blood pressure, including miRs-143, -145, -133, -21- and -1^17^. These miRNAs were examined because they regulate vascular smooth muscle cell differentiation and function, although how these miRNAs contribute to hypertension etiology requires further evaluation. A recent study found that miR-9 and miR-126 levels are decreased in PBMCs in untreated hypertensive patients. miR-9 and miR-126 expression levels were correlated with 24-hour mean pulse pressure in hypertensives and their expression levels in PBMCs may serve as a marker for target-organ damage. miR-9 expression was also positively correlated with left ventricular mass index^18^. Several miRNAs, including members of the miR-27 and miR-30 miRNA families, are abundantly expressed in microvascular endothelial cells and target mRNAs in hypertension-related pathways *in vitro*. These include hypertension-related mRNAs previously identified in GWAS analysis, such as *SH2B3* and *PLEKHA7*^19^.

We hypothesized that there is differential mRNA and miRNA expression between hypertensive AA and white women and healthy controls. Using microarray analysis, coupled with miRNA target prediction, we surveyed gene expression in PBMCs isolated from AA and white, hypertensive and normotensive women with the goal of identifying relevant gene-miRNA expression differences in hypertension-related pathways. We identified novel mRNA-miRNA pairs differentially expressed by race and/or hypertension status that have not previously been associated with hypertension. Additionally, we tested miRNA targets in primary endothelial cells to verify the functional relationship between significantly changed mRNA-miRNA pairs identified in PBMCs. We provide evidence that miRNAs contribute to differential mRNA expression levels in female hypertensives, which may aid in identifying potential new biomarkers to further explore health disparities in hypertension, specifically in AA females.

## Results

### Gene Expression Changes by Race and Hypertension

**Figure 1:**
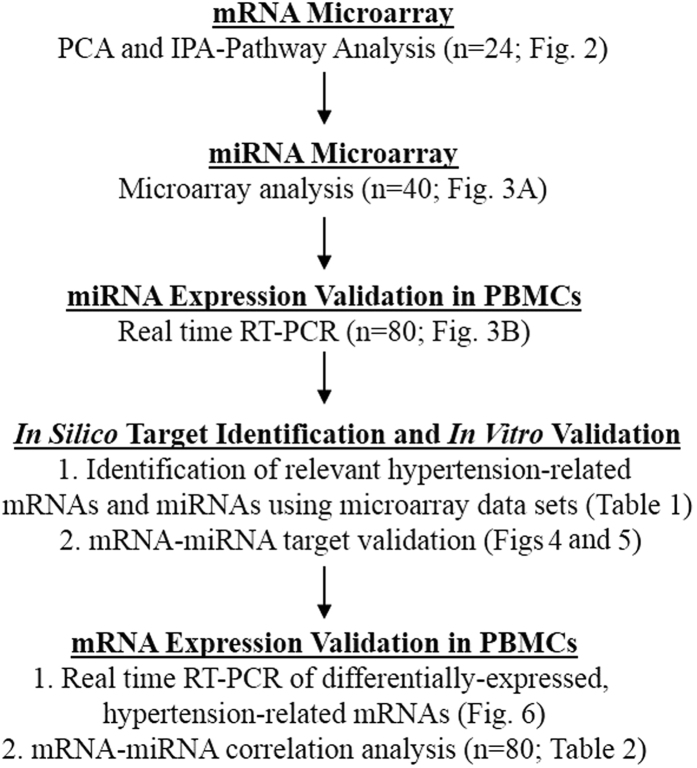
Diagram of study work flow.

**Figure 2:**
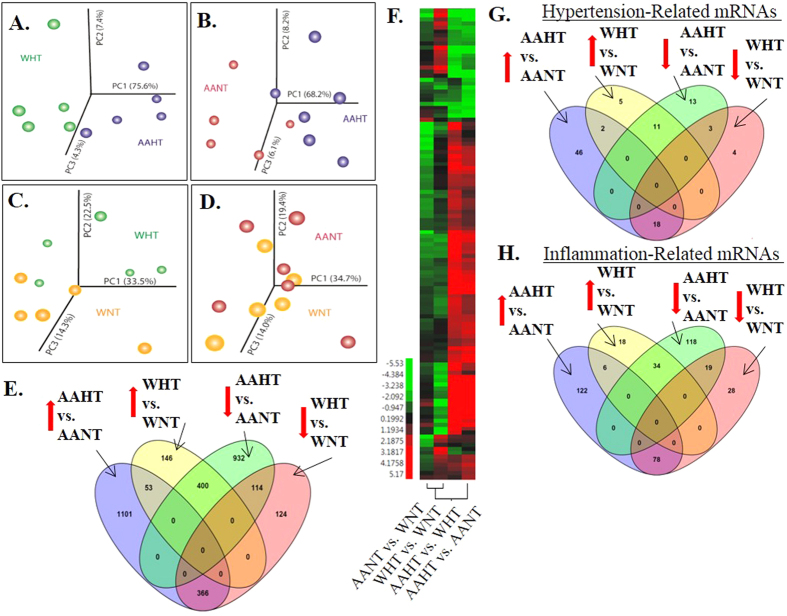
Global changes in gene expression by race and hypertension. Total RNA was isolated from PBMCs from African American (AA) or white (W) age-matched females who were normotensive (NT) or hypertensive (HT). (**A** thru **D**) Gene expression was assessed using microarray and differences in expression by either race or presence of hypertension were analyzed by PCA (n = 6/group). (**E**) Venn diagram of the total number of significantly upregulated (up arrow) or downregulated (down arrow) mRNAs for each comparison. Each comparison highlights significant changes in hypertensives compared with normotensive racial controls or changes observed in AA hypertensives compared with white hypertensives (AAHT vs. WHT) or AA normotensives compared with white normotensives (AANT vs. WNT). (**F**) Heat map of significantly-changed, hypertension-related mRNAs identified using IPA. Red (up) and green (down) indicate relative Z-ratio for each comparison. (**G**,**H**) Venn diagrams of significantly upregulated or downregulated mRNAs in the hypertension-related gene set (**G**) or inflammation-related gene set (**H**). AANT, AA normotensive; AAHT, AA hypertensive; WNT, white normotensive; WHT, white hypertensives.

The overall workflow of this study is shown in Fig. 1. We examined mRNA expression by microarray from PBMCs from 24 age-matched females divided into four subgroups: white normotensives (WNT), white hypertensives (WHT), African American normotensives (AANT), and African American hypertensives (AAHT; n = 6/group; Supplementary Table S1). mRNA expression was analyzed by Principle Component Analysis (PCA), which here demonstrates the differences between global gene expression profiles between two groups. We found a high degree of separation when comparing AAHT with WHT (PC1 75.6%; Fig. 2A) or AAHT with AANT (PC1 68.2%; Fig. 2B) and to a smaller degree when comparing WHT with WNT (PC1 33.5%; Fig. 2C) or AANT with WNT (PC1 34.7%; Fig. 2D), indicating a clear effect of race and hypertension on transcript profiles. We identified 3,554 mRNAs significantly and differentially-expressed (pairwise z-test p-values ≤ 0.05, absolute value of Z ratio ≥1.5-fold, fdr ≤0.30) when stratifying by race and presence of hypertension, or when comparing AANTs with WNTs or AAHTs with WHTs (see methods for definition of hypertension). Many genes were significantly changed in more than one comparison (AAHT with AANT or WHT; WHT with WNT; AANT with WNT; see Supplementary Excel File 1 for full gene lists). When comparing within AAs, 1,101 mRNAs were elevated specifically in AAHT compared with AANT, and 932 mRNAs were significantly decreased (Fig. 2E). Of the 270 mRNAs significantly altered when comparing WHT with WNT, 146 were elevated and 124 were decreased (Fig. 2E). Interestingly, only 167 mRNAs exhibited similar expression differences in both white and AA hypertensives while 766 mRNAs were differentially-expressed in both races but in opposite directions. These results indicated each race has a unique subset of genes differentially expressed due to hypertension status and a majority of shared genes are expressed reciprocally (Fig. 2E), suggesting the regulation of gene expression in hypertension may be influenced by race.

The mRNA expression data was imported into Ingenuity Pathway Analysis (IPA) to match against curated hypertension-related gene sets and 118 mRNAs were significantly- and differentially-expressed between groups in our cohort (Fig. 2F, Supplementary Excel File 1). 46 mRNAs were elevated in AAHT compared with AANT, and 13 mRNAs were significantly down (Fig. 2G). Of the 9 mRNAs significantly altered when comparing WHT with WNT, 5 were elevated and 4 were downregulated (Fig. 2G). We observed 5 mRNAs exhibiting similar expression patterns between AAHT and WHT, while 29 mRNAs were differentially-expression in opposite directions (Fig. 2G). Similar expression patterns were observed between AAHT and WHT in the IPA-curated, inflammation-related gene set consisting of 455 mRNAs (Fig. 2H; Supplementary Excel File 1) and within distinct pathways such as the renin-angiotensin and nitric oxide signaling pathways (Supplementary Fig. S1). Together, these results also suggest that most gene expression differences in hypertension-related pathways are specific to each race and expressed in the opposite direction.

### miRNA Changes by Race and Hypertension

**Figure 3:**
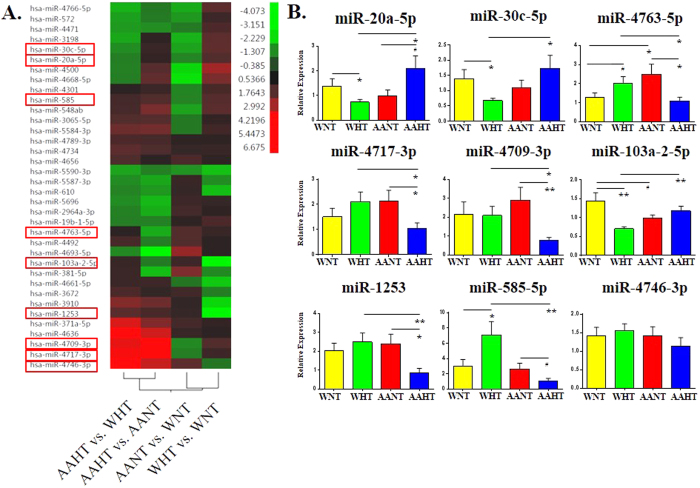
miRNA levels in PBMCs are differentially expressed by race and/or hypertension status. (**A**) Heat map of all 36 significantly-changed miRNAs in PMBCs identified by microarray (n = 10/group). Red (up) and green (down) indicate relative fold-changes for each comparison. Red boxes indicate which individual miRNAs were PCR-validated. (**B**) miRNAs were validated in an expanded cohort (n = 20/group) using RT-qPCR with miRNA-specific primers, after exclusion of outliers. Histograms represent the mean ± SEM. *P < 0.05, **P < 0.01, ^#^*P* < 0.09; Student’s t-test.

Considering the important gene regulatory role of miRNAs in the cardiovascular system^9^,^10^,^11^,^12^, we profiled miRNA expression from PBMCs from 40 individuals, including those previously profiled for mRNA expression. Each of the four subgroups (AAHT, AANT, WHT, and WNT) were expanded to ten individuals (Supplementary Table S2) and miRNA expression was evaluated using a miRNA microarray. 36 miRNAs were significantly and differentially-expressed either by race, presence of hypertension, or both (absolute value fold-change ≥1.5, Wilcoxon-Mann-Whitney test p-value ≤ 0.05, fdr ≤0.30; Fig. 3A). Differential miRNAs in AAHT vs. AANT clustered together with differences between AAHT and WHT; whereas differences in WHT vs. WNT resembled more closely miRNA expression differences when comparing AANT to WNT (Fig. 3A). These results were comparable with the mRNA differences observed between the same comparisons (Fig. 2).

Out of these 36 miRNAs, expression levels of nine individual miRNAs (outlined in red boxes in Fig. 3A) were validated in an expanded cohort (n = 20/subgroup; Supplementary Table 3) using real-time, quantitative PCR (RT-qPCR) with miRNA-specific primers (Fig. 3B). We chose to investigate these nine miRNAs from our microarray screen because they either had a large and significant fold-change in our analysis (e.g. miRs-4709-3p, -4717-3p, 4746-3p, -1253, -103a-2-5p) or there were race-specific expression differences (miRs -30c-5p, -20a-5p, -585-5p, -4763-5p). Additionally, we wanted to include miRNAs with known associations to diseases in the vasculature systems (miR-20a-5p, miR-30c-5p, and miR-103a-2-5p)^20^,^21^,^22^,^23^. We also sought to investigate novel miRNAs that have no known targets, as their functionality may be important to hypertension etiology and/or health disparities and have only recently been cataloged by next-generation sequencing (miR-4763-5p, miR-4709-3p, and miR-4717-3p)^24^.

Aside from miR-4746-3p, which was not significantly altered in any comparison (AAHT vs. AANT; WHT vs. WNT; AAHT vs. WHT; AANT vs. WNT), we observed significant differences in miRNA expression in at least one comparison for each of the remaining eight miRNAs (Fig. 3B). Several miRNAs, including miR-4717-3p, miR-4709-3p, miR-4763-5p, and miR-1253 had decreased expression in AAHT compared with both AANT and WHT, but they were similar in WHT compared with WNT (Fig. 3B). miR-20a-5p, miR-30c-5p, and miR-103a-2-5p were significantly decreased in WHT compared with WNT but were significantly increased in AAHT compared with WHT; suggested these three miRNAs may be influenced by race in hypertension. Both miR-585-5p and miR-4763-5p were increased in WHT compared to WNT and were decreased in AAHT when compared with either WHT or AANT (Fig. 3B). As many of the hypertensives in our cohort are on antihypertensive medication, we evaluated whether these medications or statin use affected miRNA expression. The use of anti-hypertensive medication did not significantly affect the expression of any of the miRNAs, but statin use was associated with a significant decrease in miR-20a-5p expression in treated hypertensives compared to untreated (Supplementary Tables S5 and S6).

To identify novel targets for each of the eight validated miRNAs differentially-expressed in PMBCs by race or hypertension status, we used the DIANA-microT algorithm^25^ to generate a list of predicted mRNA targets for each miRNA (Table 1, Columns I and II; see Supplementary Excel File 2 for individual miRNA target lists). We next matched the predicted targets for each miRNA with our mRNA microarray dataset of 3,554 significantly- and differentially-expressed mRNAs to identify all possible and relevant gene targets expressed in our dataset. We identified hundreds of possible miRNA targets differentially-expressed in our cohort for each miRNA, with the exception of miR-585-5p which was predicted to target only 38 mRNAs (Table 1, Column III).

To further parse down relevant predictions, we matched the above predictions with a manually-curated list of 1,264 unique, hypertension-related genes. This list was combined from 21 different IPA-generated gene sets after the removal of overlapping genes and known aliases, including disease-associated networks and regulatory pathways involved in hypertension pathology and cardiovascular function (see Methods). These data included genes from our inflammation- and hypertension-related gene sets (Fig. 2G,H) as well as pathway genes involved with renin-angiotensin signaling, nitric oxide signaling, cellular adhesion, and actin cytoskeletal signaling, among many others (listed in Table 1, bottom). The complete list of all genes in each gene set can be found in Supplementary Excel File 2. We identified a subset of genes for each miRNA which were predicted to target genes in hypertension-related pathways and are differentially expressed by race and/or hypertension. Most of the eight miRNAs are predicted to target >100 mRNAs, with the exception of miR-4763-5p and miR-585-5p, which are predicted to target 46 and 7 mRNAs, respectively (Table 1, Column IV).

### Hypertensive miRNA Target Validation

**Figure 4:**
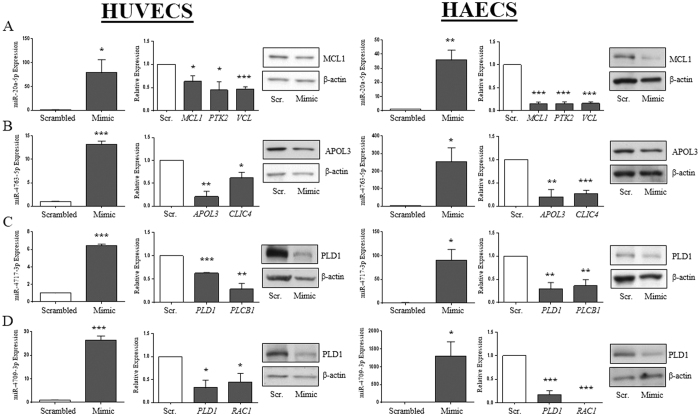
Hypertension and race-associated miRNA target validation. HUVECs (left) and HAECs (right) were transiently transfected with precursor mimics of miR-20a-5p (**A**), miR-4763-5p (**B**), miR-4717-3p (**C**), or miR-4709-3p (**D**). miRNAs and their predicted mRNA targets were quantified by RT-qPCR. miRNA levels in transfected cells are compared to scrambled control. Histograms for each individual mRNA are compared to scrambled control. Representative immunoblots of target protein levels from at least 3 independent experiments. Histograms represent the mean ± SEM. *P < 0.05; **P < 0.01; ***P < 0.001; Student’s T-test.

Five miRNAs from our dataset were further evaluated *in vitro* to functionally validate our bioinformatic prediction modeling. miR-20a-5p and miR-30c-5p were chosen because each miRNA has an identified role in regulating pathways associated with diseases in the vascular system, but neither have an identified role in essential hypertension^20^,^21^,^23^. We chose miRs -4763-5p, -4717-3p, and -4709-3p as they have not been previously associated with hypertension or any other cardiovascular disease and have no known targets, as previously mentioned. Mimics for each individual miRNA were over-expressed in human umbilical vein endothelial cells (HUVECs; Fig. 4A–D, Supplementary Fig. S2, left panels) and RNA was isolated for gene expression analysis using microarray. Hundreds of mRNAs were significantly decreased (≥1.5-fold) in the presence of each miRNA mimic (Table 1, Column V) and between 5–10% of these mRNAs were also differentially-expressed in PBMCs (Table 1, Column VI). Further parsing these data, we observed that each of miR-20a-5p, -30c-5p, -4763-5p, -4717-3p, and 4709-3p are predicted to target 28, 14, 8, 28, and 41 mRNAs in our hypertension-related gene set and which were also differentially-altered in PBMCs and downregulated in HUVECs (Table 1, Column VII; see Supplementary Excel File 2 for list of genes).

Individual mRNA targets for each miRNA were chosen for *in vitro* target validation in HUVEC-transfected cells. mRNA targets were chosen using the following criteria: predicted as a target by both the DIANA-microT and IPA algorithms *and* either (1) significantly and differentially-expressed >1.5-fold in our PMBC microarray screen or (2) significantly repressed >1.5-fold in the HUVEC microarray screen or (3) a combination of both 1 and 2. The miR-20a-5p mimic repressed HUVEC expression of *MCL1, PTK2,* and *VCL* target mRNAs as analyzed by RT-qPCR (Fig. 4A, left) and MCL1 protein levels via immunoblotting. miR-4763-5p significantly repressed expression of *APOL3* and *CLIC4* mRNA and APOL3 protein levels (Fig. 4B, left). miR-4717-3p and miR-4709-3p both repressed *PLD1* mRNA and protein and miR-4717-3p repressed target *PLCB1* mRNA and miR-4709-3p repressed *RAC1* mRNA (Fig. 4C,D, left). miR-30c-5p repressed expression of target *PDE5A* mRNA (Supplementary Fig. S2, left). Similar results for each miRNA:mRNA pair were also observed *in vitro* in primary human aortic endothelial cells (HAECs) when transfected with individual miRNA mimics, thus confirming these interactions are not cell-line specific (Fig. 4A–D, Supplementary Fig. S2).

**Figure 5:**
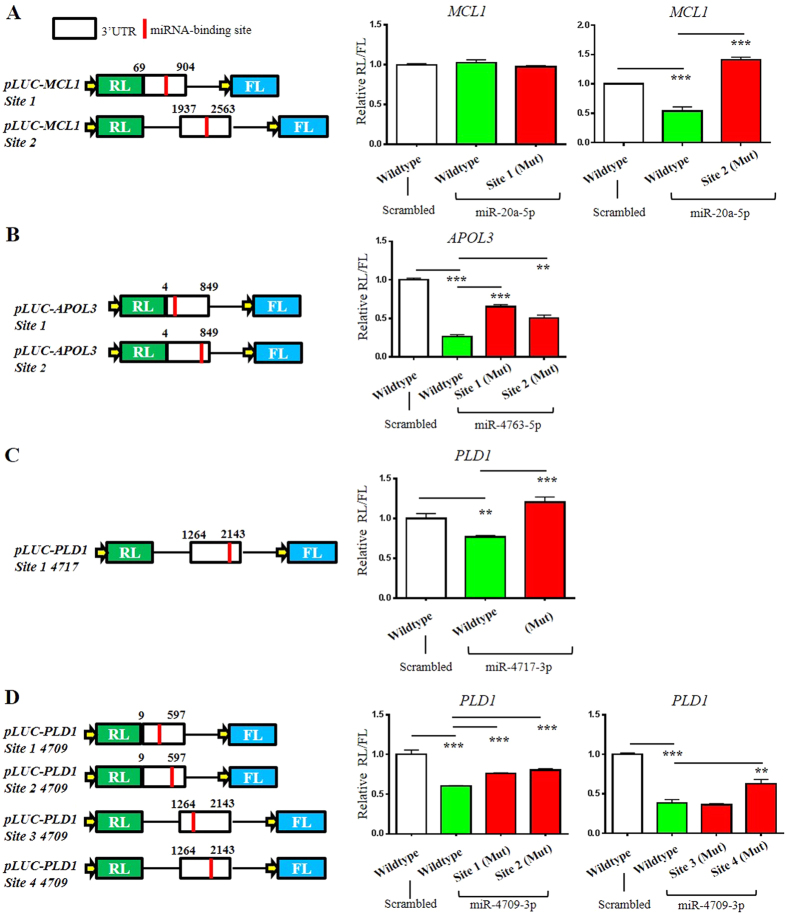
Analysis of miRNA binding sites within mRNA targets using reporters. 3′ UTR fragments from target mRNAs containing the miRNA binding sites were cloned downstream of a *Renilla* luciferase (RL) reporter (Panels **A**–**D**, left). Each plasmid also contains a Firefly luciferase (FL) reporter, which serves as a transfection control. Above each construct, the base pairs of the cloned 3′ UTRs are indicated relative to the start of each mRNA’s 3′ UTR sequence. Red bars indicate the approximate location of each miRNA binding site (see Supplemental Fig. S3 for detailed miRNA binding sites). HeLa cells were transfected with each luciferase reporter plasmid containing the partial wild-type 3′ UTRs of *MLC1* (A), *APOL3* (**B**), or *PLD1* (**C**,**D**) and either a scrambled miRNA control or indicated miRNA mimic. The ratio of RL/FL activity is shown and each wild-type plasmid was normalized and compared with the scrambled control. MicroRNA seed sequences were mutated (mut) for the indicated sites (see Supplemental Fig. S3 for specific mutations) and luciferase activity was measured as above and normalized to the scrambled control. Histograms represent the mean ± S.D. of three independent experiments. **P < 0.01, ***P < 0.001; Student’s t-test.

To further address the functional relationship between each miRNA:mRNA pair, we generated dual-luciferase reporter constructs for each target mRNA in which both mRNA and protein levels were repressed by miRNA mimics in endothelial cells. The partial 3′ UTRs for *MCL1, APOL3*, and *PLD1,* each containing predicted miRNA binding sites, were cloned downstream of a luciferase reporter plasmid (Fig. 5A–D, left panels). Luciferase constructs containing the wild-type 3′ UTR sequences were co-transfected into HeLa cells with either a scrambled control or the appropriate precursor miRNA mimic. miR-20a-5p significantly repressed the *pLUC-MCL1 Site 2* 3′ UTR (Fig. 5A), miR-4763-5p repressed the *pLUC-APOL3* 3′UTR (Fig. 5B), and the *pLUC-PLD1* reporter activity was repressed by both miR-4717-3p (Fig. 5C) and miR-4709-3p (Fig. 5D). These data confirm miRNA-mediated translation repression of the target mRNAs. We performed site-directed mutagenesis (SDM) on each binding site seed sequence (see Supplementary Fig. S3 for specific changes) to confirm the sequence specificity of each 3′UTR/miRNA binding site. The *MCL1* and *APOL3* 3′ UTRs contain two miR-20a-5p and miR-4763-5p binding sites, respectively. miR-20a-5p binding site 2 and both miR-4763-5p binding sites within each 3′ UTR increased reporter activity when mutated and compared with the wild-type plasmid (Fig. 5A,B), indicating that these sites are indeed functional. The *PLD1* 3′ UTR contains one functional miR-4717-3p binding site (Fig. 5C) and four predicted miR-4709-3p binding sites, of which sites 1, 2, and 4 are functional when compared with the wild-type plasmid (Fig. 5D). Together, these results confirm the specificity of the binding of each miRNA with its respective mRNA target.

### Hypertension and Race-associated mRNA targets in PMBCs

**Figure 6:**
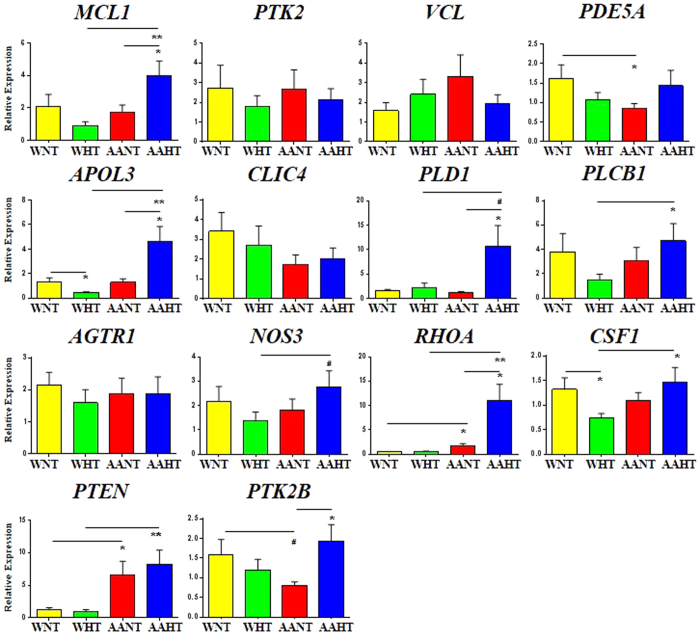
mRNA levels in PBMCs are differentially expressed by race and/or hypertension status. 14 mRNAs identified in the microarray and subsequent miRNA target analysis were validated in an expanded cohort (n = 20/group) using RT-qPCR with mRNA-specific primers, after exclusion of outliers. Histograms represent the mean ± SEM. *P < 0.05, **P < 0.01, ^#^*P* < 0.09; Student’s t-test.

Expression levels of mRNA candidates that were identified in our bioinformatic screen and assessed and validated as miRNA targets *in vitro* were quantified by RT-qPCR in PBMCs from an expanded hypertension cohort (n = 18–20/group, after removal of outliers; Supplementary Table S3). Additionally, we also quantified additional mRNAs associated with hypertension pathology that were significantly changed in at least one comparison in our screen, including *RHOA, PTEN,* and *PTK2B*. (Figure 2F,E; Supplementary Excel File 1). We also included known hypertension-related genes, including *AGTR1, NOS3,* and *CSF1* (Fig. 6). We observed that several mRNAs were significantly increased in PBMCs in AAHT compared with AANT, including *MCL1, APOL3, PLD1, RHOA,* and *PTK2B* (Fig. 6). Additionally, with the exception of *PLD1* and *PTK2B*, each of these genes were also significantly increased in AAHT when compared with WHT (Fig. 6). *PLCB1, CSF1*, and *PTEN* were also significantly increased in AAHT compared with WHT (Fig. 6). Few of these genes had significantly altered expression in WHT compared with WNT except *APOL3* and *CSF1,* which were both decreased (Fig. 6). Only *PDE5A* and *PTEN* were differentially-expressed by race in AANT compared with WNT. There were no significant differences in *PTK2¸VCL, CLIC4, AGTR1,* or *NOS3* mRNA abundance between any subgroup (Fig. 6). Additionally, the use of anti-hypertensive medication or statins did not influence mRNA levels when comparing hypertensives taking medication to those without (Supplementary Tables S7 and S8).

### Correlation of miRNA:mRNA Pairs

To further assess each miRNA:mRNA pair, we correlated the relative expression levels of each miRNA with their respective mRNA targets and the other validated mRNAs from PMBCs in our expanded cohort (n = 80; Table 2). We found that miR-4763-5p expression levels negatively correlated with *APOL3* mRNA levels in all individuals (Table 2). A significant, negative correlation was also observed in normotensive individuals of both races (Supplementary Table S9). We observed a negative trend between miR-4763-5p and *APOL3* when comparing only whites, only AAs, and in all hypertensives (Supplementary Tables S10–12). miR-4717-3p expression levels were significantly and negatively correlated with *PLD1* and *PLCB1* mRNA levels in all individuals (Table 2). miR-4717-3p and *PLD1* were significantly negatively correlated in all hypertensives and in AAs, but not whites (Supplementary Tables S10–12) and miR-4717-3p and *PLCB1* were significantly negatively correlated in all individuals (Table 2), as well as in all normotensives and in whites, but not AAs (Supplementary Tables S9–12). miR-4709-3p and *PLD1* were not significantly correlated in any of the comparisons tested (Table 2 and Supplementary Tables S9–12). These results provide further support that these miRNAs may play an important role in racial differences in hypertension.

## Discussion

We assessed for the first time global mRNA and miRNA expression levels in normotensive and hypertensive AA and white women to identify novel genes associated with essential hypertension. Our screen identified >2,000 unique, differentially-expressed mRNAs in AAs between hypertensives and controls, whereas only ~250 mRNAs were differentially-expressed and unique in whites (Fig. 2). We found that a large majority of significantly-changed mRNAs that were common between AA and white hypertensives were differentially-expressed in opposite directions, especially in hypertension-related pathways and gene sets including inflammation (Fig. 2) and the nitric oxide and renin-angiotensin signaling pathways (Supplementary Fig. S1). These data show there are significant gene expression differences in hypertension between AA and white women and suggest that molecular and genetic factors attributed to hypertension may differ by race.

Interestingly, when comparing AANT and WNT, we observed a significant decrease in phosphodiesterase 5A (*PDE5A*) mRNA levels (Fig. 6). Given that PDE5A is an important enzyme that regulates vasodilation and constriction^26^, these data suggest that race may affect gene expression levels and possibly confer predisposition to hypertension. Several genes involved in pathways related to cell adhesion and the actin cytoskeleton, including *PTK2B, RHOA*, and *PTEN* were significantly elevated in AAHT (Fig. 6). These pathways regulate vascular stiffness and response to shear stress and may affect endothelial vasoconstriction predominantly in AAs versus whites. We observed that *RHOA* is significantly higher in AANT vs. WNT. These results are similar to previous findings comparing *RHOA* expression in endothelial cells from healthy AAs and whites^5^ and suggest *RHOA* may be important in hypertension predisposition and pathology in AAs. Future studies examining this hypothesis are warranted, especially considering these pathways are not typically targeted by anti-hypertensive medications.

We identified 36 differentially-expressed miRNAs associated with hypertension and race (Fig. 3). In a cohort of European males and females, including smokers, investigators examined miRNA expression differences in PMBCs from hypertensive patients. They identified higher expression of miR-1 and miR-21 and decreased expression of miR-143, miR-145, and miR-133a in hypertensives^17^ and the same group later observed decreased levels of miR-9 and miR-126 in PMBCs in hypertensives^18^. We did not observe significant differential-expression in any of these miRNAs, perhaps because our cohort was comprised of AA and white female non-smokers only. We also did not observe differences in expression of other previously-identified, hypertension-related miRNAs such as miR-155^27^ or miR-181a^19^, which may be due to the fact that miRNA discovery was performed first in those studies in endothelial cells, not in PBMCs as reported here.

We identified several miRNAs with no known association to hypertension, including miRs-4763-5p, -4717-3p, and -4709-3p. miR-4763-5p is significantly repressed in AAHT compared to AANT, as well as WHT, and was the only miRNA of the nine which we found differentially-expressed between AANT and WNT (Fig. 3B). These differences suggest a strong racial- and hypertension-association for miR-4763-5p. Little is known about miR-4763-5p or its molecular targets. Here, we identified apolipoprotein L3 (*APOL3*) as a novel target of miR-4763-5p. APOL3 is a member of the apolipoprotein L gene family and may have a role in the metabolism and/or shuttling of lipids in the endothelium. *APOL3* expression can be induced by the inflammatory cytokine TNF-α and is elevated in atherosclerotic aortas^28^. In our cohort, *APOL3* expression levels were inversely correlated with miR-4763-5p in PBMCs in all individuals and in all normotensives, suggesting that miR-4763-5p is a primary regulator of *APOL3* expression. Increased expression of *APOL3* in AAHTs may be in response to a chronic low grade inflammatory state that may be present in AA females. This may contribute to endothelium dysfunction and increased blood pressure, possibly affecting predisposition to hypertension-related diseases such as atherosclerosis.

miR-4717-3p and miR-4709-3p expression levels are both significantly decreased in AAHT yet remained unchanged in whites, indicating that these two miRNAs may be specifically involved in hypertension physiology in AAs. miR-4717-3p regulates programmed cell death-1 (PD1)^29^ but miR-4709-3p has no confirmed targets. We identified phospholipase D1 (*PLD1*) as a novel target of both miRNAs. PLD1 modulates lipid signaling by catalyzing the hydrolysis of phosphatidylcholine into choline and the signaling molecule phosphatidic acid, which regulates multiple downstream pathways implicated in hypertension including cytoskeletal organization and inflammation^30^. PLD1 is upregulated in response to IL-1β stimulation in chronic autoimmune arthritis^31^ and PLD1 protein is regulated by RHOA^32^, which has an important role in hypertension pathophysiology^33^. Both *PLD1* and *RHOA* mRNA were elevated in AAHT, but were unchanged in whites. Furthermore, miR-4717-3p expression levels were negatively correlated with *PLD1* in our cohort and the negative correlation was strongest in AAs (Table 2 and Supplemental Table S12). These results suggest that pathways involved with PLD1 signaling and miR-4717-3p regulation may be important in inflammation and hypertension risk, particularly in AAs. Assessment of their specific role in disease as well as any potential as a therapeutic targets may clarify direct mechanisms related to blood pressure regulation and physiology.

We found that miR-20a-5p regulates the expression of *MCL1*, and both genes are differentially expressed by race in hypertension. miR-20a-5p is involved with inflammatory signaling in pulmonary hypertension^21^ and this is the first study identifying an association with essential hypertension. Myeloid cell leukemia sequence 1 (MCL1) is a member of the BCL-2 anti-apoptotic gene family and is frequently over-expressed in many cancers^34^. We observed that *MCL1* mRNA is elevated in AAHT compared with AANT and WHT, and decreased in WHT compared with WNT. Consistent with our findings in AAs, male AA prostate cancer patients have higher levels of MCL1 mRNA and protein compared to white males^35^. That study also identified miR-133a as a negative regulator of *MCL1*^35^. miR-133a has previously been associated with hypertension^17^; however, miR-133a expression was not differentially-expressed in our cohort, suggesting that additional regulatory factors (e.g. miR-20a-5p) contribute to *MCL1* regulation.

We chose to initially profile mRNA and miRNA expression patterns from PBMCs for a number of reasons. First, PBMCs are a readily obtainable biospecimen from volunteers. Second, previous studies indicated that elevated white blood cell count in women is associated with hypertension^36^ and a predictor of cardiovascular events^37^. Thirdly, elevated levels of neutrophils and decreased levels of lymphocytes are correlated with increased blood pressure in AAs^38^. The mechanistic link between subtypes of white blood cells and blood pressure regulation is still largely unknown. However, data indicates that there exists cross-talk between blood mononuclear cells and the vascular layer by a number of factors, including miRNAs^10^,^11^. Our analysis is limited in that it is an observational assessment of mRNA and miRNA expression in PBMCs and analysis of gene expression in specific white blood cell subtypes was not performed. It should be noted that several of the novel miRNA:mRNA pairs we found to be differentially-expressed in PBMCs are also co-expressed and functional in primary endothelial cells. Additional studies are needed to identify if these differentially-expressed pathways are also found to be differentially-expressed in endothelial cells isolated from hypertensive individuals. Considering the crosstalk between immune cells and the vascular endothelial layers, it is possible that gene expression in PBMCs can serve as a biomarker for gene expression in the endothelium or that differentially-expressed miRNAs observed in our cohort are actively signaling to the endothelium and exuding a functional impact on endothelial function. Future work lies in further characterizing the functional roles of these genes with respect to hypertension pathology. At this time our results do not provide direct evidence as to whether differentially-expressed pathways and miRNA:mRNA pairs identified here are a cause or consequence of hypertension status. Further evaluation of their direct roles in hypertension etiology and blood pressure regulation, especially with regard to different races, are required and an understanding of the mechanisms controlling differential miRNA expression will further enhance our understanding of this phenomena.

We cannot rule out the possibility that additional mechanisms contribute to the regulation of differential mRNA expression in PBMCs of AA and white hypertensive women. Ethnicity is associated with the inheritance of allelic variants in several inflammatory cytokines, including IL-1, IL-6, and IL-18, that are linked with increased levels of cytokine production, however, no association between ethnicity and allelic variants of TNFα have been observed^39^,^40^,^41^. African American women in particular are more likely to carry allelic variants associated with elevated levels of the pro-inflammatory cytokines IL-1 and IL-6 and variants associated with decreased production of anti-inflammatory IL-10^41^. miR-155 is a known regulator of both endothelial nitric oxide synthase 3 (eNOS)^27^ and angiotensin II type 1 receptor (AGTR1)^42^, which regulate blood vessel relaxation and constriction, respectively. There is a functional polymorphism (rs5189) located within the miR-155 binding site in the 3′ UTR of *AGTR1* that disrupts miR-155 regulation of *AGTR1* expression, thereby increasing AGTR1 expression and risk for cardiac-related complications^42^,^43^,^44^. In AAs with hypertension, the presence of this polymorphism may increase the risk of chronic kidney disease^45^. In our cohort, we did not observed changes in miR-155 or *AGTR1* expression levels, but there was a significant increase in *NOS3* (which encodes eNOS) gene expression in AAHTs compared with WHTs (Fig. 6). Variants have been identified in AAs in other genes identified in our study, including *APOL3*^46^. Future work lies in identifying variants in the 3′ UTRs of the identified mRNAs in the miRNA:mRNA pairs discussed in this study and whether they functionally influence gene expression in our cohort or in the pathways that are differentially-expressed between AA and white hypertensive women.

Importantly, this is the first global analysis of differential gene expression in AA and white hypertensive women. Using microarray screening and miRNA-prediction modeling, we identified novel miRNAs and mRNA targets with significant differential-expression in hypertension that were influenced by race. Although there is no general consensus that race is a major determinant in response to anti-hypertension medication, particularly, mono-therapeutic strategies^47^,^48^, our analysis identifies gene expression differences that could have important implications in the progression and treatment of hypertension for different races. We also provide evidence that miRNAs functionally contribute to differentially-expressed genes in the context of hypertension and racial disparities. Further exploration of these novel miRNA:mRNA pairs and their role in hypertension etiology is warranted, particularly with respect to functional contributions as a cause or consequence of chronic states of elevated blood pressure. Ultimately this may help identify more suitable targets for preventive and therapeutic approaches to hypertension, bringing to fruition personalized targeted approaches to age-related and disparate diseases.

## Materials and Methods

### Study participants and peripheral blood mononuclear cell isolation

Fasting blood samples were obtained from participants in the Healthy Aging in Neighborhoods of Diversity across the Life Span (HANDLS) study of the National Institute on Aging Intramural Research Program (NIA IRP), National Institutes of Health (NIH). HANDLS is a longitudinal, epidemiological study based in Baltimore, MD that seeks to identify the causes of age-associated health disparities by examining the relationship between race, health, socio-economic factors, dietary influences, and other factors^49^. The HANDLS participants are AA and white between the ages of 30–64 at baseline and residing in Baltimore, MD. The Institutional Review Board of the National Institute of Environmental Health Sciences, NIH, approved this study and all participants provided written informed consent. All experiments were performed in accordance with relevant guidelines and regulations.

The demographics of the HANDLS participants used in this study are presented in Supplementary Tables S1–3. For this sub-cohort, we chose age-matched, AA and white females who were either normotensive (NT) or hypertensive (HT) at the baseline wave of the HANDLS study. Hypertension was defined as an average SBP ≥140 mmHg or DP ≥90 mmHg or use of previously prescribed antihypertensive medications (diuretics, beta blockers, ACE inhibitors, angiotensin II blockers, calcium channel blockers, or vasodilators) and/or prior diagnosis of hypertension. We excluded participants with documented Hepatitis B, Hepatitis C, or human immunodeficiency virus infection and current or former smokers. Isolation of PBMCs was performed as previously described^50^.

Blood pressure and pulse were measured in both arms while seated after a 5 minute rest and averaged. Respiratory rate was reported as total number of breaths per minute and taken after a 5 minute rest. Body mass index (weight [kg]/height [m^2^]) was calculated from measured height and weight. Total cholesterol, high density lipoprotein (HDL), low-density lipoprotein (LDL), triglycerides, and hsCRP were measured at Quest Diagnostics (Chantilly, Virginia). Serum cytomegalovirus IgG positivity was measured by ELISA (Genway Biotech, San Diego, CA). Data was collected during a structured medical history interview and a physical examination.

### Cell culture, transfection, and RNA isolation

Primary human umbilical vein endothelial cells (HUVECs) and primary human aortic endothelial cells (HAECs) were purchased from Lonza (Walkersville, MD). HeLa cells were grown in Dulbecco’s modified Eagle’s medium (DMEM) supplemented with 10% FBS and 1% sodium pyruvate. HUVECs were grown in EBM supplemented with the EGM SingleQuot Kit (Lonza). HAECs were grown in EMB-2 supplemented with the EGM-2 SingleQuot Kit (Lonza). Cells were transfected with Pre-miR miRNA Precursors for hsa-miR-20a-5p, -4763-5p, -4717-3p, -4709-3p, -30c-5p or scrambled negative control using Lipofectamine 2000 (Life Technologies). Total RNA from HUVECs and HAECs was isolated using TRIzol (Life Technologies). Isolation of total RNA from PBMC for microarray and validation analysis was performed using the miRVana miRNA Isolation Kit with enrichment for small RNAs (Life Technologies). RNA quality was measured by a Nanodrop 2000 and Agilent Bioanalyzer.

### Microarray analysis and target prediction

Gene expression in PBMCs and HUVECs was analyzed by microarray using the Illumina Beadchip HT-12 v4 (San Diego, CA). RNA was prepared and labeled according to the manufacturer’s protocol. Raw signal data were filtered by the detection p-values and Z-score normalization to obtain normalized probe signals. One-way ANOVA tests (p-value ≤ 0.05) on the sample groups were used to eliminate the probes/genes with larger variances within each group. Genes with pairwise z-test p-values ≤ 0.05, absolute value of Z ratio ≥1.5-fold, and an fdr ≤0.3 were considered significant.

PBMC miRNA expression was assessed by microarray using the Exiqon miRCURY LNA microRNA 7^th^ gen-human-mouse-rat Array (Vedbaek, Denmark) and RNA was prepared and labeled according to the manufacturer’s protocol. Microarray data was analyzed using non-parameterized statistical methods since miRNA expression patterns did not follow a Gaussian distribution. The original fluorescent signal was globally normalized using the median of each sample. The probe levels were filtered using the confident interval (CI) for each probe within four technical repeats. The marked probes with values outside the CI were removed as outliers and the median probe value was recalculated. We performed a one-way independent Kruskal-Wallis test to examine the data variance across each sample group and the consistency of global probe levels were compared to control with a p-value cutoff ≤0.05. Each significant comparison had an absolute value fold-change ≥1.5, Wilcoxon-Mann-Whitney test p-value ≤0.05, and an fdr ≤0.30 as the correction error control cutoff. The microarray data has been submitted to GEO (Super Series Number: GSE75672).

Ingenuity Pathways Analysis (IPA; Ingenuity Systems, Redwood City, CA) was used to visualize changes in mRNA expression in our cohort. IPA network analysis utilizes a curated knowledge base of known functional interactions, miRNA prediction using the TargetScan^51^ and DIANA-Tarbase^52^, and previously identified protein functions to algorithmically infer biochemical interactions. We curated 21 gene sets and pathways related to cardiovascular function and inflammatory disease to identify as many genes as possible in a hypertension-related context (see Supplementary Excel File 2 for complete gene lists for each pathway). We selected pathways with known associations with hypertension etiology (e.g renin-angiotensin signaling) or manually built connected pathways from hypertension related genes according to gene function using IPA’s default settings and pathway builder functions (i.e. hypertension pathway gene list). Significance of gene expression changes, functions, and pathways were calculated using the right-tailed Fisher’s Exact Test using a fold-change cutoff of >1.5-fold. A manually-curated list of 1,264 mRNAs was generated from these 21 IPA gene sets after the removal of overlapping genes between pathways and known alias (see Table 1 and Supplementary Excel File 2). This curated list was matched against target genes for each miRNA as predicted by DIANA-microT v5.0^25^,^53^ using a prediction threshold of 0.5. The DIANA-microT algorithm was used in addition to IPA because every publicly available miRNA prediction algorithm weighs prediction criterion differently (reviewed in Saito *et al*.^54^) and DIANA-microT predicts miRNA binding sites within protein coding sequences. This strategy was employed to increase the potential identification of all possible regulatory miRNAs in our bioinformatics workflow for each significantly changed mRNA in our analysis and before target validation and confirmation *in vitro*.

### RT-qPCR analysis

Total RNA was transcribed into cDNA using the QuantiMiR RT Kit (Systems Biosciences, Mountain View, CA). For mRNA, real-time RT-qPCR reactions were performed with 2x SYBR Green Master Mix and gene specific primers (Supplementary Table S4) on a 7900HT Fast Real-Time PCR System or 7500 Real-Time PCR System according to manufacturer’s protocols (Life Technologies). For miRNAs, forward primers were designed to be the exact sequence of the mature miRNAs, as listed in miRBase (http://www.mirbase.org). miRNA primers are listed in Supplementary Table S4 and a universal reverse primer was supplied with the QuantiMir RT Kit. In PBMCs, miRNA expression was normalized to the average of *U6*, miR-147a, and miR-574-5p and mRNA levels were normalized to the average of *GAPDH* and *ACTB*. In HUVECs and HAECs, miRNA expression was normalized to the average of *U6* and *U24* and mRNA levels were normalized to the average of *GAPDH* and *ACTB*. All gene expression levels were calculated using the 2^−ΔΔCt^ method^55^.

### Western blot analysis

HUVECs and HAECs were washed twice with cold PBS and lysed in 2X Laemmli sample buffer. Protein levels were assessed by immunoblotting with anti-APOL3 or anti-MCL1 (Abcam, Cambridge, MA), anti-PLD1 (Santa Cruz, Dallas, TX) or anti-actin (Santa Cruz) antibodies.

### 3′ UTR Luciferase reporter assays

cDNA fragments containing most of the 3′ UTR of human *APOL3* and partial 3′ UTRs of human *MCL1* and *PLD1* were PCR-amplified using specific primers with XhoI and NotI adapted ends (primer sequences are in Supplementary Table S4). After NotI and XhoI digestion, PCR products were cloned downstream of the *Renilla* open reading frame in the psiCHECK2 reporter plasmid from Promega (Madison, WI). Each psiCHECK2-3′ UTR reporter construct containing mutations or deletions in the predicted miRNA binding site seed sequences (summarized in Supplementary Fig. S3) were created using the QuikChange site-directed mutagenesis kit (Agilent, Santa Clara, CA) according to the manufacturer’s protocol. HeLa cells were co-transfected with 25 or 50 ng of the indicated 3′-UTR construct with either 50 nM scrambled control or the corresponding miRNA precursor mimic (Life Technologies). Forty-eight hours later, *Renilla* (RL) and *Firefly* (FL) activities were measured using the Dual-Luciferase reporter assay system (Promega) according to the manufacturer’s instructions. FL served as an internal transfection control and the ratio of RL to FL for the wild-type and mutated 3′ UTRs co-transfected with miRNA mimics was normalized to the wild-type plasmid with scrambled control for each construct.

### Statistical analysis

The Student’s *t*-test was used when comparing two groups, unless otherwise indicated. miRNA and mRNA levels in cohort PBMCs were examined for Gaussian distribution by measuring skewness, kurtosis, and with a visual inspection of histogram plots. Outliers for each gene were excluded from analysis using Grubb’s test with an alpha = 0.05. The one-tailed Pearson test was used for correlation analysis. A *p*-value of < 0.05 was considered statistically significant.

## Additional Information

**How to cite this article**: Dluzen, D. F. *et al*. Racial differences in microRNA and gene expression in hypertensive women. *Sci. Rep.* **6**, 35815; doi: 10.1038/srep35815 (2016).

## Supplementary Material

Supplementary Information

Supplementary Excel File 1 (Dataset 1)

Supplementary Excel File 2 (Dataset 2)

## Figures and Tables

**Table 1:** Summary of miRNA:mRNA target prediction.

I. microRNA	II. DIANA-microT predicted targets (^#^of genes)	III. ^#^Predicted targets differentially-expressed in PBMCs	IV. ^#^Predicted targets within IPA* gene sets & differentially-expressed in PBMCs	V. Total ^#^genes significantly repressed ≥1.5-fold by miRNA over-expression in HUVECs	VI. ^#^Predicted targets both repressed ≥1.5-fold by miRNA over-expression in HUVECs and differentially expressed in PBMCs	VII. ^#^Predicted targets repressed ≥1.5-fold by miRNA over-expression in HUVECs, differentially expressed in PBMCs, and within IPA*gene sets
miR-20a-5p	4,712	834	195	1,130	89	28
miR-30c-5p	3,724	669	150	833	45	14
miR-4763-5p	1,539	231	46	1,569	41	8
miR-4717-3p	3,632	628	153	1,661	114	28
miR-4709-3p	4,682	791	175	1,958	207	41
miR-103a-2-5p	4,031	669	147	N/A	N/A	N/A
miR-1253	4,729	710	158	N/A	N/A	N/A
miR-585-5p	255	38	7	N/A	N/A	N/A

*IPA gene lists (^#^genes/list): Inflammation-related (455), hypertension-related (118), renin-angiotensin (50) and nitric oxide signaling (44), focal adhesion kinase (77), actin cytoskeleton (84), left ventricle dysfunction (88), PI_3_K-Akt (58), hypertension pathway (136), VEGF (41), inflammation of the artery (54), atherosclerosis (45), blood flow (42), calcium signaling (47), Endothelin-1 (32), IL-6 (51) and NF-kB signaling (75), reactive oxygen species in macrophages (51), STAT3 pathway (23), blood pressure (129), CVD targets (18); 1,264 total genes. The individual genes for each gene set are listed in Supplemental Excel File 2.

**Table 2:** Correlation of PBMC expression levels of all miRNA and mRNA pairs in all individuals.

**Gene**	miR-20a-5p	miR-30c-5p	miR-4763-5p	miR-4717-3p	miR-4709-3p	miR-103a-2-5p	miR-1253	miR-585-5p
*MCL1*	0.41	0.44	−0.23*	−0.40***	−0.24*	0.53	−0.46***	−0.38***
*PTK2*	0.30	0.56	−0.13	−0.27**	0.04	0.46	−0.37***	−0.30**
*VCL*	0.08	0.06	−0.05	−0.14	0.10	−0.04	−0.15	0.08
*PDE5A*	0.41	0.42	−0.21*	−0.30**	−0.25*	0.55	−0.22*	−0.20*
*APOL3*	0.33	0.48	−0.23*	−0.23*	−0.15	0.31	−0.32**	−0.25*
*CLIC4*	0.28	0.30	0.03	−0.34**	−0.31**	0.24	−0.34**	−0.35**
*PLD1*	0.24	0.42	−0.14	−0.25*	−0.11	0.03	−0.27**	−0.23*
*PLCB1*	0.32	0.31	−0.17	−0.30**	−0.17	0.60	−0.41***	−0.32**
*AGTR1*	0.11	0.05	0.10	−0.14	−0.08	0.01	−0.09	−0.20*
*NOS3*	0.39	0.46	−0.07	−0.40***	−0.21*	0.32	−0.38***	−0.38***
*RHOA*	0.30	0.30	−0.12	−0.17	−0.14	0.08	−0.27*	−0.20*
*CSF1*	0.17	0.28	−0.22*	−0.16	−0.09	0.24	−0.05	−0.14
*PTEN*	0.54	0.38	0.02	−0.35**	−0.03	0.39	−0.38***	−0.36**
*PTK2B*	0.33	0.23	−0.13	−0.34**	−0.32**	0.30	−0.36***	−0.35**

One-tailed Pearson r correlation values are indicated. *P < 0.05, **P < 0.01, ***P < 0.001.
