# Supplementary material for: Racial differences in microRNA and gene expression in hypertensive women
Source: Sci Rep. 2016 Oct 25;6:35815. doi: 10.1038/srep35815 (PMC5078799; doi:10.1038/srep35815)
Supplement: Supplementary Information [file srep35815-s1.pdf]

SUPPLEMENTAL MATERIAL

Racial differences in microRNA and gene expression in hypertensive women

Douglas F. Dluzen[1], Nicole Noren Hooten[1], Yongqing Zhang[2], Yoonseo Kim[1], Frank E. Glover[1], Salman M. Tajuddin[1], Kimberly D. Jacob[1ŧ], Alan B. Zonderman[1], and Michele K. Evans[1*]

[1]Laboratory of Epidemiology and Population Sciences; [2]Laboratory of Genetics and Genomics; National Institute on Aging, National Institutes of Health, Baltimore, MD 21224, USA

[ŧ]Current address Department of Biology, Franklin and Marshall College, Lancaster, PA 17604-3003

Table S1: Demographics for mRNA microarray

Characteristic	White normotensive (WNT) n=6	White hypertensive (WHT) n=6	African American normotensive (AANT) n=6	African American hypertensive (AAHT) n=6
Age, y	55.3 ± 3.20	55.3 ± 3.20	53.8 ± 3.49	55.2 ± 3.92
BMI	31.3 ± 2.99	33.2 ± 4.40	28.0 ± 5.98	34.0 ± 8.10
Total cholesterol, mg/dL	326 ± 30.5	227 ± 70.2	177 ± 38.6	194 ± 50.6
HDL, mg/dL	62 ± 13	53.8 ± 10.1	58.3 ± 17.9	51.3 ± 9.91
LDL, mg/dL	136 ± 19.2	117 ± 19.3	104 ± 36.8	119 ± 52.0
Triglycerides, mg/dL	137 ± 74.7	281 ± 407	73.8 ± 25.9	**119 ± 23.5***
hsCRP, mg/L	3.67 ± 2.87	3.79 ± 3.08	6.93 ± 11.5	20.0 ± 18.7
Systolic BP, mmHg	121 ± 12.2	122 ± 24.7	115 ± 6.31	**137 ± 17.2***
Diastolic BP, mmHg	74.8 ± 8.01	70.8 ± 7.41	67.0 ± 8.22	76.3 ± 8.07
Resp. Rate	13.3 ± 3.27	13.7 ± 1.97	16.8 ± 3.13	15.7 ± 3.45
Pulse	75.7 ± 4.80	73.6 ± 10.4	72.3 ± 10.2	71.0 ± 9.70
Heart disease, N (% Total)	0 (0.0)	0 (0.0)	0 (0.0)	1 (16.7)
Menopausal/Post-menopause, n (% Total)	6 (100.0)	4 (66.7)	5 (83.3)	6 (100.0)
Estrogen Therapy, n (% Total)	0 (0.0)	1 (16.7)	0 (0.0)	0 (0.0)
rx Statins, n (% Total)	0 (0.0)	2 (33.3)	1 (16.7)	3 (50.0)
CMV Serum+, n (% Total)	5 (83.3)	5 (83.3)	5 (83.3)	5 (83.3)
Diabetes, n (% Total)	0 (0.0)	1 (16.7)	0 (0.0)	1 (16.7)
Below Poverty, N (% Total)	2 (33.3)	1 (16.7)	3 (50.0)	1 (16.7)
rx Hypertension, n (% Total)	0 (0.0)	**3 (50.0)†**	0 (0.0)	**5 (83.3)†**

*,P<0.05; AAHT vs. AANT, Student's T-test

†,P<0.001; HTN vs. NHT; Fisher's Exact Test

WNT = white normotensive; WHT = white hypertensive; AANT = African American normotensive; AAHT = African American hypertensive; HTN = all hypertensives; NHT= non hypertensives

Table S2: Demographics for miRNA microarray

Characteristic	White Non-hypertensive (WNHT) n=10	White Hypertensive (WHT) n=10	African American Non-hypertensive (AANT) n=10	African American Hypertensive (AAHT) n=10
Age, y	54.7 ± 2.91	54.9 ± 3.18	54.9 ± 3.18	54.9 ± 3.18
BMI	28.9 ± 4.37	**34.3 ± 6.55***	26.6 ± 5.33	**34.7 ± 7.13†**
Total cholesterol, mg/dL	222 ± 26.4	209 ± 59.2	168.8 ± 36.3	190.6 ± 38.1
HDL, mg/dL	59.5 ± 14.0	52.5 ± 13.4	60.2 ± 15.7	57.0 ± 11.0
LDL, mg/dL	135 ± 19.4	**111 ± 19.1***	94.1 ± 35.8	113 ± 39.8
Triglycerides, mg/dL	136 ± 63.7	226 ± 313	72.4 ± 19.8	**102 ± 29.5†**
hsCRP, mg/L	3.49 ± 2.86	7.37 ± 10.1	4.33 ± 9.18	14.4 ± 16.2
Systolic BP, mmHg	115 ± 12.3	118 ± 19.6	114 ± 7.28	**132 ± 17.4†**
Diastolic BP, mmHg	71.7 ± 7.83	70.7 ± 6.22	65.6 ± 6.88	**77.4 ± 9.36†**
Resp. Rate	13.8 ± 2.91	14.6 ± 2.07	15.9 ± 3.02	16.2 ± 3.46
Pulse	71.4 ± 7.6	73.0 ± 8.55	74.0 ± 10.3	73.2 ± 10.9
Heart disease, N (% Total)	0 (0.0)	2 (20.0)	0 (0.0)	1 (10.0)
Menopausal/Post-menopause, n (% Total)	8 (80.0)	7 (70.0)	9 (90.0)	9 (90.0)
Estrogen Therapy, n (% Total)	0 (0.0)	1 (10.0)	0 (0.0)	0 (0.0)
rx Statins, n (% Total)	0 (0.0)	**4 (40.0)‡**	1 (10.0)	**3 (30.0)‡**
CMV Serum+, n (% Total)	7 (10.0)	8 (80.0)	7 (10.0)	9 (90.0)
Diabetes, n (% Total)	0 (0.0)	**2 (20.0)‡**	0 (0.0)	**2 (20.0)‡**
Below Poverty, N (% Total)	2 (20.0)	3 (30.0)	3 (30.0)	2 (20.0)
rx Hypertension, n (% Total)	0 (0.0)	**7 (70.0)§**	0 (0.0)	**7 (70.0)§**

*, $P<0.05$; WHT vs. WNT, Student's T-test

†, $P<0.05$; AAHT vs. AANT; Student's T-test

‡, $P<0.05$; HTN vs. NHT; Fisher's Exact Test

§, $P<0.001$; HTN vs. NHT; Fisher's Exact Test

WNT = white normotensive; WHT = white hypertensive; AANT = African American normotensive; AAHT = African American hypertensive; HTN = all hypertensives; NHT= non hypertensives

Table S3: Demographics for validation cohort

Characteristic	White Non-hypertensive (WNT) n=20	White Hypertensive (WHT) n=20	African American Non-hypertensive (AANT) n=20	African American Hypertensive (AAHT) n=20
Age, y	54.3 ± 2.52	56.5 ± 2.91	54.7 ± 2.91	56.0 ± 2.78
BMI	30.1 ± 5.6	34.1 ± 8.5	28.2 ± 4.94	**34.4 ± 6.2***
Total cholesterol, mg/dL	214 ± 31.0	200 ± 51.2	185 ± 34.4	192 ± 37.3
HDL, mg/dL	54.7 ± 13.3	51.3 ± 12.7	58.0 ± 15.3	61.4 ± 12.9
LDL, mg/dL	131 ± 31.3	112 ± 29.2	110 ± 33.0	112 ± 26.1
Triglycerides, mg/dL	144 ± 66.2	134 ± 69.6	82.6 ± 22.9	92.5 ± 31.8
hsCRP, mg/L	4.11 ± 4.02	7.06 ± 8.14	4.31 ± 6.98	10.9 ±12.6
Systolic BP, mmHg	116 ± 13.2	125 ± 22.0	116 ± 10.5	**133 ± 15.8***
Diastolic BP, mmHg	71.6 ± 7.12	72.4 ± 8.58	69.2 ± 8.37	75.7 ± 9.68
Resp. Rate	14.3 ± 2.21	15.2 ± 2.80	16.2 ± 3.13	16.1 ± 3.06
Pulse	72.9 ± 8.30	73.2 ± 8.76	73.6 ± 10.8	74.1 ± 8.52
Heart disease, N (% Total)	0 (0.0)	2 (10.0)	0 (0.0)	1 (5.0)
Menopausal/Post-menopause, n (% Total)	15 (75.0)	17 (85.0)	18 (90.0)	19 (95.0)
Estrogen Therapy, n (% Total)	1 (5.0)	1 (5.0)	0 (0.0)	0 (0.0)
rx Statins, n (% Total)	0 (0.0)	**5 (25.0)†**	2 (10.0)	**7 (35.0)†**
CMV Serum+, n (% Total)	12 (60.0)	15 (75.0)	16 (80.0)	18 (90.0)
Diabetes, n (% Total)	1 (5.0)	**5 (25.0)‡**	1 (5.0)	**3 (15.0)‡**
Below Poverty, N (% Total)	3 (15.0)	7 (35.0)	7 (35.0)	6 (35.0)
rx Hypertension, n (% Total)	0 (0.0)	**13 (65.0)§**	0 (0.0)	**12 (60.0)§**

*, $P<0.05$; AAHT vs. AANT, Student's T-test

†, $P<0.01$; HTN vs NHT, Fisher's Exact Test

‡, $P<0.001$; HTN vs NHT, Fisher's Exact Test

§, $P<0.05$; HTN vs. NHT, Fisher's Exact Test

WNT = white normotensive; WHT = white hypertensive; AANT = African American normotensive; AAHT = African American hypertensive; HTN = all hypertensives; NHT = all normotensives

Table S4: Primer sequences for RT-qPCR

Gene	Forward Primer	Reverse Primer
U6	CGCAAGGATGACACGCAAATTC	
miR-20a-5p	TAAAGTGCTTATAGTGCAGGTAG	
miR-4763-5p	CGCCTGCCCAGCCCTCCTGCT	
miR-30c-5p	TGTAAACATCCTACACTCTCAGC	
miR-103a-2-5p	AGCTTCTTTACAGTGCTGCCTTG	
miR-4717-3p	ACACATGGGTGGCTGTGGCCT	
miR-4709-3p	TTGAAGAGGAGGTGCTCTGTAGC	
miR-4746-3p	AGCGGTGCTCCTGCGGGCCGA	
miR-1253	AGAGAAGAAGATCAGCCTGCA	
miR-585-5p	CTAGCACACAGATACGCCCAGA	
miR-147a	GTGTGTGGAAATGCTTCTGC	
miR-574-5p	TGAGTGTGTGTGTGTGAGTGTGT	
RNU24	TTAAACCACCAAGATCGCTGA	GGTGATGACATTTTAAAC
CSF1	GCAAGAACTGCAACAACAGC	TCACTGCTAGGGATGGCTTT
GAPDH	GCTCCTCCTGTTCGACAGTCA	ACCTTCCCCATGGTGTCTGA
ACTB	GGACTTCGAGCAAGAGATGG	AGCACTGTGTTGGCGTACAG
APOL3	AGAGAGTCAGCCCAGTGCAT	AGAGAGCATCTGCCTCATCC
PLCB1	TGCACGCCTTGCAACTCAA	ACAATAGTTGAGTCATCATCCCAC
PDE5A	ACTTGCATTGCTGATTGCTG	AGTAAAGCTGGGCAAGTGGA
PTEN	TTTAAAGGCACAAGAGGCCC	GGGAATAGTTACTCCCTTTTTGTCT
MCL1	ACAAAGAGGCTGGGATGGGT	TACTCCAGCAACACCTGCAA
VCL	GTCCGGGTTGGAAAAGAGAC	CTTGGTGCAAGCATTCTCAA
PTK2B	GATAAACTATATGGCAGGGAGGGC	CACTCTTACCCCAAACTCAGGT
PTK2	TGTGGGTAAACCAGATCCTGC	AAGCTTGACACCCTCGTTGT
NOS3	ACATCTTCAGCCCCAAACGG	GGATCAGACCTGGCAGCAAC
RHOA	GGATTCGTTGCCTGAGCAAT	GGGAACTGGTCCTTGCTGAA
PLD1	TAACGTACAGTTGCTCCGCTC	ATCACATGGACGTAAGCGGC
CLIC4	GAAACTGCCCCTTTTCCCAGA	CTGGCTTCCTTTTCAGGTCAAC
AGTR1	CGCGGGTTTGATATTTGACA	AAATACACCTGGTGCCGACT
APOL3 3’ UTR Site 1 SDM	TTATCCCCCTAATAAAATGGGTGCATTTTGTCGTGGCCTG	CAGGCCACGACAAAATGCACCCATTTTATTAGGGGGATAA
APOL3 3’ UTR Site 2 SDM	TTCAGTTAATTTTCTGTCTCTTTGGGTGCTGTATATGAGTAATGAGACTG	CAGTCTCATTACTCATATACAGCACCCAAAGAGACAGAAAATTAACTGAA
PLD1 3’ UTR Site 1 SDM (miR-4709-3p)	GCTAGACATTGGCTGCATAAATGCCGATCAGAGAAGAATAAGGAGATT	AATCTCCTTATTCTTCTCTGATCGGCATTTATGCAGCCAATGTCTAGC
PLD1 3’ UTR Site 2 SDM (miR-4709-3p)	GGGCATCCCATGTAACCATGCCGAATCTTGAAGCAGCATTAC	GTAATGCTGCTTCAAGATTCGGCATGGTTACATGGGATGCCC
PLD1 3’ UTR Site 3 SDM (miR-4709-3p)	ATCGGCTGCTTGCTTCTTTCTAGAACAACCCAAATGAGAG	CTCTCATTTGGGTTGTTCTAGAAAGAAGCAAGCAGCCGAT
PLD1 3’ UTR Site 4 SDM (miR-4709-3p)	CTTGGGCAATGATAAAGTGCCGAGAGAGGCCAACAATGGG	CCCATTGTTGGCCTCTCTCGGCACTTTATCATTGCCCAAG
MCL1 3’ UTR Site 1 SDM	TGGTGATAAACTAGGCTAATAATAAGAATCATGGAAACCAAGCC	GGCTTGGTTTCCATGATTCTTATTATTAGCCTAGTTTATCACCA
MCL1 3’ UTR Site 2 SDM	CAGATCTTAAGATTAATTAAAAACTACATACCGTGCTTTTAGGTCCTTAGAGATACATGATA	TATCATGTATCTCTAAGGACCTAAAAGCACGGTATGTAGTTTTTAATTAATCTTAAGATCTG
PLD1 3’ UTR SDM (miR-4717-3p)	GTAGGGGGAGACAGACAAAGTCATAAATACAAAATATCAGAGGGTTCA	TGAACCCTCTGATATTTTGTATTTATGACTTTGTCTGTCTCCCCCTA

Table S5. Influence of anti-hypertension medication on miRNA levels in hypertensives

Gene	Average Expression in Untreated Hypertensives (n=15; ± S.E.M.)	Average Expression in Treated Hypertensives (n=25; ± S.E.M.)	P-Value (Treated vs. Untreated)*
miR-20a-5p	1.60 ± 0.45	2.46 ±0.86	1
miR-30c-5p	1.36 ± 0.46	2.73 ± 1.26	0.675
miR-4763-5p	2.02 ± 0.39	1.81 ±0.34	0.693
miR-4717-3p	1.47 ± 0.28	2.11 ± 0.42	0.283
miR-4709-3p	1.42 ± 0.35	2.05 ± 0.51	0.557
miR-103a-2-5p	1.01 ± 0.13	1.25 ± 0.18	0.655
miR-1253	1.80 ± 0.37	2.71 ± 0.60	0.277
miR-585-5p	2.87 ± 1.37	5.80 ± 1.57	0.201
miR-4746-3p	1.32 ± 0.21	1.40 ± 0.21	0.811

*Mann-Whitney T-test

Table S6. Influence of statins on miRNA levels in hypertensives

Gene	Average Expression in Untreated Hypertensives (n=28; ± S.E.M.)	Average Expression in Treated Hypertensives (n=12; ± S.E.M.)	P-Value (Treated vs. Untreated)*
miR-20a-5p	2.61 ± 0.77	1.05 ± 0.47	**0.046**
miR-30c-5p	2.57 ± 1.13	1.38 ± 0.59	0.232
miR-4763-5p	1.58 ± 0.31	2.61 ± 0.47	0.065
miR-4717-3p	1.77 ± 0.39	2.10 ± 0.33	0.608
miR-4709-3p	1.77 ± 0.47	1.90 ± .040	0.244
miR-103a-2-5p	1.27 ± 0.17	0.91 ± 0.07	0.526
miR-1253	2.21 ± 0.50	2.73 ± 0.66	0.563
miR-585-5p	4.81 ± 1.45	4.47 ± 1.53	0.891
miR-4746-3p	1.16 ± 0.17	1.85 ± 0.29	**0.038**

*Mann-Whitney T-test

Table S7. Influence of anti-hypertension medication on mRNA levels in hypertensives

Gene	Average Expression in Untreated Hypertensives (n=15; ± S.E.M.)	Average Expression in Treated Hypertensives (n=25; ± S.E.M.)	P-Value (Treated vs. Untreated)*
MCL1	2.11 ± 0.58	3.10 ± 1.22	0.576
PTK2	1.55 ± 0.43	2.38 ± 0.63	0.346
VCL	3.69 ±1.57	2.93 ± 0.88	0.801
PDE5A	1.58 ± 0.43	2.29 ± 0.73	0.451
APOL3	2.86 ± 1.09	3.33 ± 1.16	0.328
CLIC4	8.35 ± 3.79	4.11 ± 2.15	0.451
PLD1	6.04 ± 3.80	10.9 ± 4.03	0.127
PLCB1	2.49 ± 0.74	2.94 ± 1.03	0.264
AGTR1	3.67 ± 1.40	3.66 ± 1.47	0.635
NOS3	2.27 ± 0.71	2.96 ± 1.15	0.635
RHOA	3.95 ± 1.78	5.34 ± 1.87	0.911
CSF1	1.41 ± 0.34	1.43 ± 0.37	0.516
PTEN	10.7 ± 4.18	7.81 ± 3.79	0.371
PTK2B	2.55 ± 1.08	2.86 ± 1.56	0.503

*Mann-Whitney T-test

Table S8. Influence of statins on mRNA levels in hypertensives

Gene	Average Expression in Untreated Hypertensives (n=28; ± S.E.M.)	Average Expression in Treated Hypertensives (n=12; ± S.E.M.)	P-Value (Treated vs. Untreated)*
MCL1	2.52 ± 0.59	3.20 ± 2.31	0.384
PTK2	1.83 ± 0.45	2.58 ± 0.95	0.422
VCL	3.42 ± 0.93	2.75 ± 1.58	0.295
PDE5A	1.77 ± 0.47	2.63 ± 1.18	0.565
APOL3	2.95 ± 0.89	3.77 ± 1.94	0.522
CLIC4	5.53 ± 2.35	6.12 ± 3.69	0.507
PLD1	8.66 ± 3.54	10.04 ± 5.47	0.829
PLCB1	2.73 ± 0.82	2.87 ± 1.35	0.69
AGTR1	4.11 ± 1.46	2.62 ± 0.71	0.526
NOS3	2.83 ± 1.04	2.42 ± 0.85	0.965
RHOA	4.23 ± 1.42	6.19 ± 3.04	0.941
CSF1	1.60 ± 0.35	0.96 ± 0.25	0.408
PTEN	8.91 ± 3.50	8.81 ± 4.84	0.871
PTK2B	2.74 ± 1.39	2.76 ± 1.34	0.545

*Mann-Whitney T-test

Table S9. Correlation between PBMC expression levels of all miRNA and mRNA pairs in all normotensive individuals.

Gene	miR-20a-5p	miR-30c-5p	miR-4763-5p	miR-4717-3p	miR-4709-3p	miR-103a-2-5p	miR-1253	miR-585-5p
MCL1	0.53	0.66	-0.14	-0.37*	-0.15	0.57	-0.47**	-0.39*
PTK2	0.51	0.85	-0.15	-0.31*	0.11	0.55	-0.40**	-0.37*
VCL	0.16	0.19	0.08	-0.11	0.20	-0.01	-0.12	-0.10
PDE5A	0.43	0.47	-0.41**	-0.36*	-0.33*	0.56	-0.18	-0.05
APOL3	0.24	0.52	-0.31*	-0.39*	-0.02	0.32	-0.30*	-0.22
CLIC4	0.51	0.44	-0.01	-0.38*	-0.36*	0.34	-0.41**	-0.38**
PLD1	0.24	0.29	0.03	-0.18	0.09	0.12	-0.22	-0.21
PLCB1	0.41	0.42	-0.17	-0.37*	-0.15	0.65	-0.45**	-0.42**
AGTR1	-0.04	-0.10	0.12	-0.08	-0.06	-0.15	-0.03	-0.02
NOS3	0.25	0.34	0.03	-0.39**	-0.15	0.39	-0.36*	-0.35*
RHOA	0.09	0.46	0.15	-0.16	0.13	0.18	-0.19	-0.28
CSF1	-0.14	0.19	-0.27	-0.05	-0.08	0.10	0.11	0.24
PTEN	0.35	0.32	0.19	-0.27	0.20	0.15	-0.35*	-0.36*
PTK2B	0.50	0.35	-0.26	-0.37*	-0.33*	0.36	-0.28*	-0.27

One-tailed Pearson r correlation values are indicated. *P<0.05, **P<0.01, ***<P<0.001

Table S10. Correlation between PBMC expression levels of all miRNA and mRNA pairs in all hypertensive individuals.

Gene	miR-20a-5p	miR-30c-5p	miR-4763-5p	miR-4717-3p	miR-4709-3p	miR-103a-2-5p	miR-1253	miR-585-5p
MCL1	0.35	0.29	-0.33*	-0.42**	-0.37*	0.57	-0.44**	-0.41**
PTK2	0.17	0.18	-0.13	-0.26	-0.29*	0.14	-0.45**	-0.34*
VCL	0.03	-0.08	-0.32*	-0.21	-0.14	-0.16	-0.21	0.22
PDE5A	0.39	0.40	0.02	-0.25	-0.18	0.60	-0.27	-0.27
APOL3	0.34	0.49	-0.25	-0.21	-0.21	0.42	-0.36*	-0.30*
CLIC4	0.12	0.14	0.09	-0.30*	-0.28*	0.11	-0.27	-0.36*
PLD1	0.21	0.43	-0.24	-0.28*	-0.21	0.10	-0.30*	-0.27*
PLCB1	0.27	0.22	-0.18	-0.21	-0.26	0.57	-0.37*	-0.31*
AGTR1	0.22	0.18	0.07	-0.21	-0.19	0.21	-0.16	-0.26
NOS3	0.48	0.56	-0.20	-0.43**	-0.32*	0.27	-0.41**	-0.42**
RHOA	0.30	0.31	-0.19	-0.19	-0.16	0.14	-0.31*	-0.26
CSF1	0.34	0.34	-0.26	-0.30*	-0.19	0.40	-0.26	-0.26
PTEN	0.66	0.44	-0.20	-0.45**	-0.37*	0.67	-0.42**	-0.42**
PTK2B	0.23	0.15	0.03	-0.30*	-0.31*	0.31	-0.43**	-0.44**

One-tailed Pearson r correlation values are indicated. *P<0.05, **P<0.01, ***P<0.001

Table S11. Correlation between PBMC expression levels of all miRNA and mRNA pairs in all white individuals.

Gene	miR-20a-5p	miR-30c-5p	miR-4763-5p	miR-4717-3p	miR-4709-3p	miR-103a-2-5p	miR-1253	miR-585-5p
MCL1	0.56	0.72	-0.36*	-0.38**	-0.34*	0.62	-0.44**	-0.32*
PTK2	0.46	0.87	-0.25	-0.30*	-0.26**	0.59	-0.43**	-0.34*
VCL	-0.14	-0.15	-0.41**	-0.25	-0.16	-0.14	-0.14	0.34
PDE5A	0.54	0.49	-0.24	-0.36*	-0.26	0.53	-0.26	-0.25
APOL3	0.40	0.49	-0.26	-0.27	-0.19	0.42	-0.11	-0.25
CLIC4	0.53	0.52	-0.06	-0.43**	-0.38*	0.35	-0.48**	-0.46**
PLD1	0.21	0.23	0.10	-0.23	-0.14	0.07	-0.33*	-0.40**
PLCB1	0.44	0.48	-0.37**	-0.39**	-0.26	0.72	-0.44**	-0.34*
AGTR1	0.01	0.02	0.13	-0.03	0.02	0.09	0.01	-0.25
NOS3	0.41	0.40	-0.19	-0.42**	-0.34*	0.44	-0.36*	-0.39**
RHOA	0.31	0.32	-0.38*	-0.42**	-0.38*	0.03	-0.28	-0.09
CSF1	-0.07	0.20	-0.29*	-0.19	-0.18	0.18	0.07	-0.05
PTEN	0.38	0.07	0.05	-0.26	-0.13	0.25	-0.23	-0.36*
PTK2B	0.56	0.40	-0.20	-0.47**	-0.40	0.33	-0.45**	-0.46**

One-tailed Pearson r correlation values are indicated. **P*<0.05, ***P*<0.01, ***<*P*<0.001

Table S12. Correlation between PBMC expression levels of all miRNA and mRNA pairs in all African American individuals.

Gene	miR-20a-5p	miR-30c-5p	miR-4763-5p	miR-4717-3p	miR-4709-3p	miR-103a-2-5p	miR-1253	miR-585-5p
MCL1	0.32	0.28	-0.18	-0.41**	-0.15	0.48	-0.45**	-0.47**
PTK2	0.23	0.40	-0.03	-0.24	0.40	0.23	-0.32*	-0.30*
VCL	0.15	0.12	0.13	-0.06	0.32	0.05	-0.13	-0.16
PDE5A	0.38	0.40	-0.17	-0.26	-0.28	0.63	-0.21	-0.27
APOL3	0.28	0.44	-0.26	-0.23	-0.14	0.31	-0.37*	-0.25
CLIC4	0.12	0.13	0.18	-0.26	-0.25	0.04	-0.25	-0.32*
PLD1	0.19	0.39	-0.20	-0.28*	-0.09	0.02	-0.27	-0.24
PLCB1	0.25	0.21	-0.04	-0.20	-0.06	0.46	-0.36*	-0.29*
AGTR1	0.15	0.07	0.09	-0.22	-0.18	-0.09	-0.17	-0.22
NOS3	0.38	0.49	0.00	-0.39**	-0.06	0.19	-0.38**	-0.41**
RHOA	0.29	0.27	-0.14	-0.19	-0.15	0.05	-0.28*	-0.18
CSF1	0.24	0.30	-0.18	-0.11	0.00	0.31	-0.11	-0.25
PTEN	0.54	0.42	0.00	-0.43**	0.06	0.57	-0.42**	-0.48**
PTK2B	0.20	0.10	-0.08	-0.20	-0.23	0.26	-0.27	-0.22

One-tailed Pearson r correlation values are indicated. **P*<0.05, ***P*<0.01, ***<*P*<0.001

Supplementary Figure S1

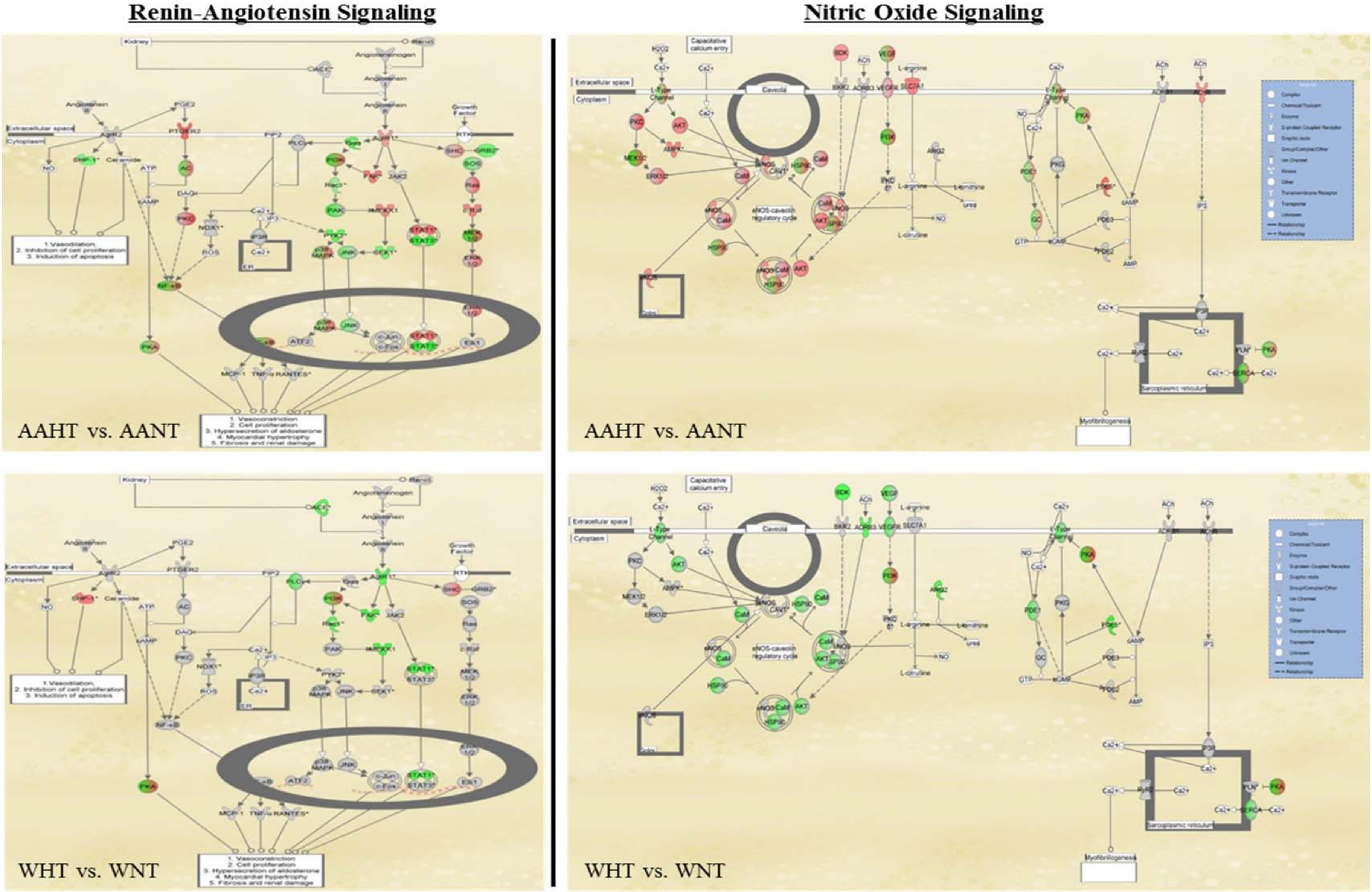

Supplementary Figure S2

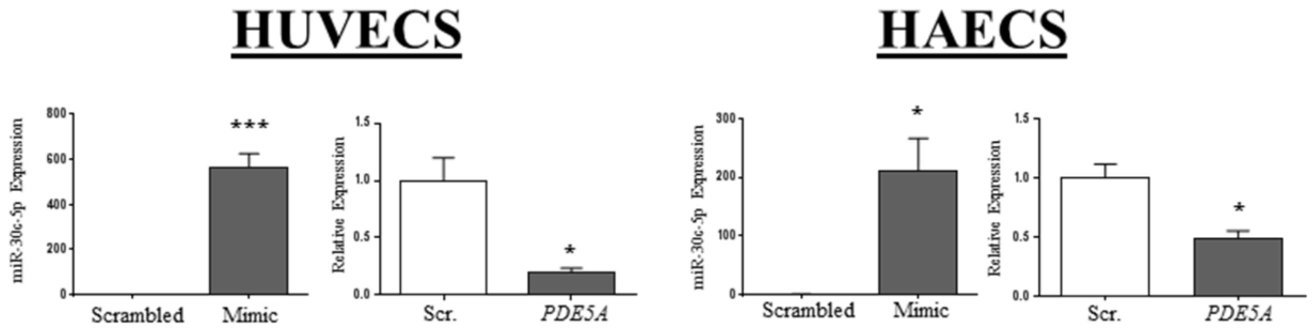

Supplementary Figure S3

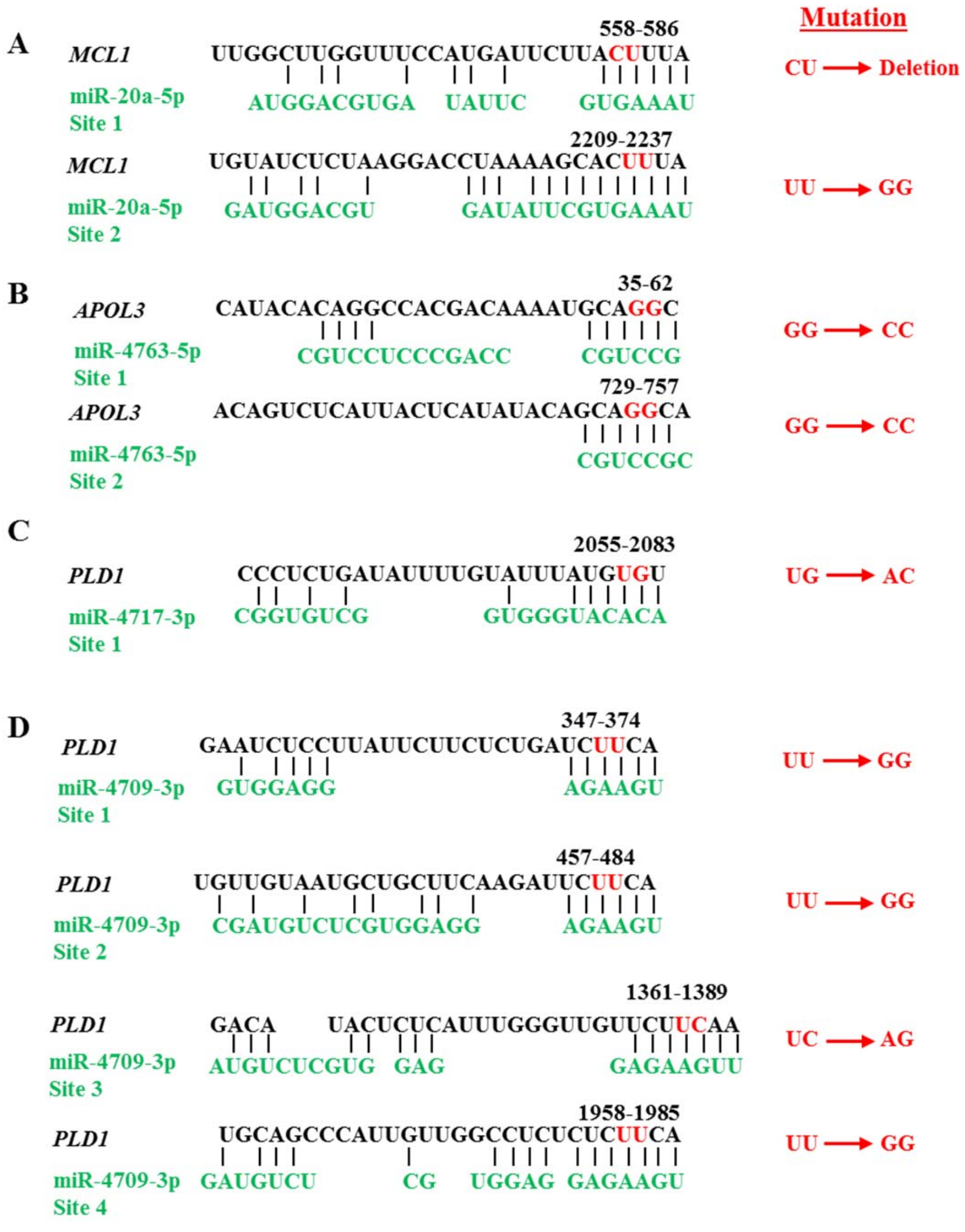

Supplemental Figure Legends

Supplementary Figure S1: Analysis of mRNA expression changes in hypertension-related gene pathways. IPA was used to visualize fold changes in mRNA expression in hypertensive African Americans (AA; Top) or whites (Bottom) in the renin-angiotensin signaling pathway (Left) or in the nitric oxide signaling pathway (Right). Expression levels are compared with normotensive controls for each race. Red indicates significantly up-regulated genes and green indicates significantly down-regulated genes.

Supplementary Figure S2: Hypertension and race-associated miRNA target validation. HUVECs (left) and HAECs (right) were transiently transfected with precursor miR-30c-5p mimic. Predicted target *PDE5A* was quantified by RT-qPCR. miRNA levels are compared to scrambled control. Histograms for *PDE5A* expression levels in the presence of miR-30c-5p are compared to scrambled control. Histograms represent the mean ± SEM. * $P<0.05$; *** $P<0.001$; Student's T-test.

Supplementary Figure S3: Point mutations introduced into predicted miRNA binding sites in target mRNA 3' UTRs. Predicted miRNA binding sites of *MCL1* (A), *APOL3* (B), *PLD1* (C & D) and their respective miRNA regulators are indicated (green). The numbers above each binding site represent the specific nucleotides within each 3' UTR, starting from the first nucleotide of the 3' UTR of each mRNA. Nucleotides (red) within each binding site seed sequence were changed using site-directed mutagenesis and changes are indicated on the right.
